# Unlocking potential in underachieving learners: self-regulation development through AI-supported e-mentoring among socioeconomically disadvantaged students

**DOI:** 10.3389/fpsyg.2026.1805629

**Published:** 2026-03-23

**Authors:** Yan Zhao

**Affiliations:** School of Biomedical Sciences, Shandong First Medical University & Shandong Academy of Medical Sciences, Jinan, Shandong, China

**Keywords:** AI-supported e-mentoring, learning engagement, self-regulated learning, socioeconomic disadvantage, underachieving students

## Abstract

Underachieving students from socioeconomically disadvantaged backgrounds often experience difficulty sustaining self-regulated learning (SRL), which contributes to persistent gaps in participation and academic achievement. This study evaluated a three-tiered AI-supported e-mentoring intervention designed to strengthen SRL through goal clarification, micro-strategy prompting, reflective coaching, and growth-oriented maintenance support. In a cluster randomized controlled trial with pre-intervention, post-intervention, and short follow-up assessments, students in the AI-supported mentoring condition showed greater gains than controls in SRL, with improvements observed in both scale-based SRL indicators and platform-based behavioral markers (e.g., planning and task completion). These gains were accompanied by better academic performance, learning engagement, and persistence. Mechanism-oriented analyses further suggested that SRL operated as a proximal pathway linking the mentoring intervention to broader learning-related outcomes. Equity-focused moderation analyses indicated a compensatory pattern, with larger benefits among students with lower baseline self-regulation and greater socioeconomic constraints. Follow-up estimates suggested that effects were largely maintained after the intensive phase, although a modest attenuation was observed. Robustness checks and missing-data diagnostics supported the stability of the findings across alternative specifications. Overall, the findings suggest that AI-supported e-mentoring, implemented with teacher oversight and safety protocols, can support SRL development and promote more sustained engagement and achievement among underachieving learners.

## 1 Introduction

Academic underachievement among students from socioeconomically disadvantaged backgrounds is not merely a consequence of insufficient knowledge acquisition, but also a persistent inequality problem shaped by uneven access to educational support and learning opportunities (Zheng and Yu, 2024). In many contexts, students from lower socioeconomic backgrounds face structural constraints in obtaining sustained academic guidance, which increases the likelihood of disengagement and long-term underachievement. At the same time, recent discussions on educational inclusion suggest that emerging AI-supported approaches may offer new possibilities for reducing access barriers, provided that they are designed with equity and support needs in mind (Cotilla Conceição and van der Stappen, 2025). Consequently, effective support for this population should extend beyond knowledge-based remediation and focus on creating psychologically supportive and scalable learning environments.

Traditional mentoring models have demonstrated certain benefits in supporting disadvantaged and underrepresented learners, especially when mentor support helps students develop stronger self-regulated learning (SRL) strategies (Tise et al., 2023). However, these manual, high-touch interventions depend heavily on sustained human involvement, making them costly and difficult to scale across time and space. As a result, the most vulnerable student populations frequently lack access to stable, individualized mentoring due to uneven resource distribution. In parallel, research on SRL interventions in online and blended environments shows that structured support can improve learning outcomes, but implementation quality and continuity remain critical for effectiveness (Guntur and Purnomo, 2024). For academically disadvantaged learners with weak self-control, the provision of technical tools alone is often insufficient to ensure meaningful learning transformation.

Within the framework of educational psychology, self-regulated learning is widely recognized as a core psychological mechanism through which latent cognitive potential is translated into observable academic outcomes. Recent evidence from educational technology research indicates that AI-based tools, including chatbots and related systems, can support SRL processes when they provide structured prompts, feedback, and strategy guidance (Guan et al., 2025; Achuthan, 2025). According to classic self-regulation models, learners sustain motivation and progress through iterative cycles of forethought, performance, and reflection (Zimmerman, 2000). Underachieving students commonly exhibit disruptions within this cycle, characterized by vague goal setting, insufficient strategic planning, weak metacognitive monitoring during task execution, and negative emotional responses during feedback stages. Importantly, self-regulation is not an innate trait but a domain-sensitive psychological process shaped through adaptive feedback and guided practice (Pintrich, 2000). Through timely cognitive guidance and external prompts, individuals can gradually develop habits of self-monitoring and strategic adjustment under supportive conditions. From this perspective, interventions targeting self-regulated learning among disadvantaged students function by constructing an externalized regulatory scaffold—effectively a “digital external brain”—that assists learners in managing higher-order cognitive regulation demands, thereby reducing cognitive load in complex learning contexts.

With the rapid development of generative artificial intelligence, new opportunities have emerged to address educational inequality, and early evidence suggests that generative AI may improve the performance of disadvantaged students when used in structured educational contexts (Brunton et al., 2025). However, the effectiveness of AI support depends strongly on how the system is designed and supervised. Recent evidence also shows that generative AI without appropriate guardrails may harm learning, highlighting the need for well-defined pedagogical boundaries, safety protocols, and human oversight (Bastani et al., 2025). To address this gap, the present study proposes and empirically evaluates an AI-supported e-mentoring model designed to examine how such a context facilitates the reconstruction of self-regulated learning cycles among underachieving students. Beyond assessing direct improvements in academic performance, this study further investigates whether AI-mediated support influences goal-related self-efficacy and metacognitive anxiety, thereby contributing to more adaptive learning outcomes.

## 2 Methods

### 2.1 Study design and procedure

A cluster randomized controlled trial was conducted, with randomization implemented at the class level to assign eligible students to either the intervention or control condition. Class-level randomization was selected because support processes for disadvantaged underachieving students are typically embedded in shared instructional routines and peer networks, which increases the risk of contamination if randomization is performed at the individual level (Campbell et al., 2012). Individual randomization could therefore produce information spillover and imitation effects that would weaken internal validity. The total study period was 12 weeks, including an 8-week intervention phase and a 4-week follow-up phase, to assess both immediate effects and short-term maintenance.

The study procedure comprised five sequential stages: participant screening and recruitment, baseline assessment, class-level allocation, post-intervention assessment, and follow-up assessment. Recruitment was initiated through school-based informational sessions. Students from socioeconomically disadvantaged backgrounds with documented low academic performance were enrolled according to predefined eligibility criteria. After written informed consent was obtained from students and their parents or guardians, baseline assessments were administered at Week 0. These assessments included psychological measures (e.g., self-regulated learning, self-efficacy, school belongingness, and academic emotion regulation) and objective or semi-objective indicators (e.g., academic performance and learning engagement records). Class-level randomization was then conducted by an independent staff member not involved in intervention delivery, and allocation results were communicated to the implementation team.

During Weeks 1–8, students in the intervention group received AI-supported e-mentoring combined with structured self-regulated learning (SRL) training, whereas students in the control group continued to receive routine school-based learning support or unguided access to learning resources. This design ensured access to basic educational services for all participants while preventing exposure to intervention mechanisms equivalent to those used in the experimental condition. Post-intervention assessments were conducted at Week 8 using measures aligned with baseline instruments. Follow-up assessments were conducted at Week 12 to examine maintenance of effects after the intensive phase. Throughout the study, missing data and reasons for attrition were systematically documented to support adherence analyses, missing-data diagnostics, and sensitivity testing.

### 2.2 Participants and context

The study was implemented in participating schools and focused on students from socioeconomically disadvantaged families. Socioeconomic vulnerability was defined using a multi-criteria approach to reduce misclassification. Classification relied primarily on school administrative records indicating financial hardship or eligibility for assistance programs and was supplemented by questionnaire-based indicators, including parental education, household learning resources, and the availability of a supportive home learning environment.

Academic underachievement was identified using objective records of persistently low performance at the class or grade level, together with teacher evaluations of learning engagement and homework completion. This combined approach was used to ensure that enrolled students exhibited sustained learning difficulties rather than temporary performance fluctuation.

Inclusion criteria required students to be within the target grade range, able to participate regularly in intervention activities and assessments during the study period, and able to access a personal digital device or a school-provided alternative. Exclusion criteria included severe learning disabilities or neurodevelopmental disorders requiring specialized educational services, inability to participate in routine learning interventions, and anticipated prolonged absence during the intervention or assessment windows.

The study was conducted in authentic school settings to enhance ecological validity and to evaluate the feasibility of AI-supported e-mentoring under real resource constraints. To accommodate the practical conditions of disadvantaged students, the intervention was designed to remain interactive while maintaining low burden and low access thresholds. Core organizational tasks (e.g., scheduling reminders and attendance follow-up) were jointly managed by homeroom teachers and trained research assistants to reduce avoidable attrition related to contextual instability.

### 2.3 Intervention

The intervention combined an AI-supported mentoring system with structured SRL training to help underachieving students develop sustainable habits of learning initiation, process monitoring, and reflective adjustment. The intervention was designed to shift students from passive task completion toward more deliberate planning, strategic control, and persistence. Across the 8-week program, students received both structured dialogic guidance and ongoing daily support, enabling continuity without imposing excessive burden.

Each weekly cycle followed a consistent behavioral sequence. The cycle began with prompts addressing students’ current learning status and perceived stress, with the aim of increasing problem awareness and preparing students for regulation-oriented planning. Students were then guided to define short-term, attainable learning goals and convert these goals into actionable plans. Goals were further decomposed into executable task units, including approximate time allocation and concrete first steps. This modular task design was especially important in resource-constrained contexts, as it reduced the likelihood of unrealistic scheduling and early abandonment.

The next phase focused on strategy support and execution guidance. The AI mentor provided personalized strategy suggestions aligned with students’ goal types and self-identified barriers, including attentional control techniques, error tracking routines, spaced repetition, and self-reward rules. Students retained autonomy in strategy selection, while repeated use of structured prompts helped stabilize behavioral patterns over time.

In the middle and later phases of the intervention, reflective practice and attribution adjustment were intensified. Students were guided to review their recent performance, identify effective strategies, and recognize contextual barriers that constrained task completion. When appropriate, the AI mentor supported attributional reframing from stable ability-based explanations toward modifiable strategic or situational factors, with the aim of strengthening perceived control and persistence. In the final phase, adaptive challenge and maintenance modules were introduced. After students established a basic SRL structure, task difficulty was gradually increased or transfer-oriented tasks were incorporated, accompanied by mastery-oriented feedback. This phase was designed to consolidate regulatory skills and support longer-term maintenance of SRL routines.

The control group received routine school-based learning support or non-mentor-style resource recommendations but did not receive structured dialogic scaffolding, reflective coaching, or personalized strategy generation. This condition was designed to preserve a meaningful comparison while minimizing overlap with the core mechanisms of the intervention.

### 2.4 Platforms, human oversight, and safety protocols

The intervention platform used a conversational AI mentor as the primary interaction interface and integrated task generation, reminder scheduling, and review logging to create a closed-loop learning support system. The platform was designed to provide structured and interpretable guidance rather than open-ended, nonspecific conversation. Dialogic interactions were organized into fixed modules that guided students through goal clarification, strategy planning, method selection, and reflective review within each interaction cycle. Core interaction content was retained as traceable learning logs to support adherence assessment and mechanism analysis.

To ensure technical reproducibility, the AI mentor was implemented using OpenAI GPT-4o via the OpenAI API in a text-only deployment mode for the present trial (OpenAI, 2024). The model configuration was fixed throughout the intervention period to reduce variation in response behavior, with temperature set at 0.3, top_p set at 1.0, and maximum output length constrained to 350 tokens per response. The platform did not enable internet browsing or external tool use, and all responses were generated solely from student input and the predefined prompt framework. Prompt construction followed a documented prompt-engineering approach emphasizing role specification, task decomposition, response constraints, and safety boundaries (White et al., 2023). Prompt versions were finalized before the trial and managed through internal version control tags (v1.0–v1.2), and no substantive prompt changes were introduced after participant enrollment. To support technical reproducibility while protecting participant privacy, representative prompt templates used in the intervention (system prompt, planning prompt, and reflection prompt) are provided in Appendix A in de-identified form.

The prompting framework was structured into a stable sequence of role guidance, planning guidance, strategy coaching, reflective review, and safety handling. The system prompt defined the AI as a school-based learning mentor for underachieving adolescents from socioeconomically disadvantaged backgrounds and explicitly required a supportive, non-judgmental, and action-oriented communication style. It also prohibited the model from providing medical advice, mental health diagnosis, psychotherapy substitutes, crisis counseling, or direct homework answers, and required referral to human support when severe emotional distress or safety risk was disclosed. The goal-planning prompt required the model to help students identify one to three realistic short-term learning goals, convert them into manageable task units, assign approximate study time, estimate difficulty, and identify a backup plan if the student’s schedule was disrupted. The strategy-support prompt instructed the model to provide two to three problem-matched strategies based on the student’s reported difficulty, such as attentional drift, procrastination, memory difficulty, confusion, or low motivation, and to explain why each strategy fit the current barrier and how the student could apply it immediately. The reflection prompt guided students to review recent learning performance without self-blame, identify what was completed, identify obstacles, and revise future plans, while also supporting attributional reframing from fixed-ability explanations toward modifiable strategic or situational factors. A dedicated safety-response template was embedded to override routine mentoring whenever the student’s text indicated self-harm ideation, severe distress, abuse risk, or inability to remain safe, in which case the system generated a brief supportive response that directed the student to seek immediate help from a teacher, parent or guardian, or school counselor and simultaneously triggered a supervisor alert. Appendix A provides representative examples of these prompt templates and their functional structure.

Because the study involved vulnerable adolescents, a human-supervised, tiered governance framework was implemented to ensure ethical and practical safety (UNESCO, 2023). At the data level, de-identification procedures were applied, and students accessed the platform using assigned study codes rather than personal names. Only data necessary for intervention delivery and research analysis were retained, and all storage and access procedures were restricted to authorized personnel. A dual-channel risk identification mechanism was established using both behavioral indicators and textual content. Repeated weeks of very low participation, persistently high self-reported stress, or interaction content containing explicit self-harm references or severe emotional distress automatically triggered alerts to designated human supervisors.

Human supervisors were appointed by the school or selected from trained members of the research team. Their responsibilities included reviewing alerts, verifying urgency, and initiating appropriate support or referral pathways according to school safeguarding procedures. To reduce the likelihood of inappropriate AI-generated guidance, the platform applied explicit guardrails at critical stages and restricted responses in high-risk domains, including medical diagnosis, psychotherapy substitution, and crisis intervention. Students were also reminded within the platform that the AI mentor was designed for learning support rather than emergency or clinical support, and they were encouraged to contact school counselors, teachers, or parents and guardians when necessary. All manual interventions and risk-management actions were documented in anonymized procedural logs, with only essential records retained in order to balance traceability and privacy protection. This combination of technical constraints and human oversight was designed to preserve scalability while meeting the safety and ethical requirements of adolescent school-based intervention research.

### 2.5 Measures and outcomes

The measurement framework was aligned with the intervention logic and included both psychological self-regulated learning (SRL) indicators and objective or semi-objective behavioral outcomes reflecting changes in academic functioning. Measures also captured implementation dosage, intervention process quality, and mechanism-relevant indicators to support interpretation and replicability (Panadero, 2017; Baker and Inventado, 2014). Unless otherwise specified, scale-based measures were collected at three time points (baseline, post-intervention, and follow-up) to evaluate both immediate effects and short-term maintenance. Academic performance and behavioral indicators were obtained from school records and platform-generated logs whenever available, thereby reducing the risk of single-source self-report bias.

Primary outcomes were defined as co-primary indicators that combined scale-based SRL outcomes with behavioral SRL markers. The scale-based SRL outcomes assessed overall SRL and key dimensions including goal setting, planning, process monitoring, adjustment capacity, strategy use, and reflective evaluation. These dimensions were selected to capture differential intervention effects across the forethought, performance, and reflection phases of the SRL cycle. To address potential social desirability bias in self-report measures, behavioral SRL markers were elevated from process indicators to co-primary outcomes. These behavioral indicators included weekly plan creation, on-time plan submission, micro-task completion rate, weekly goal attainment rate, and review submission frequency, or equivalent platform-recorded self-monitoring behaviors. For participants in the control condition, where direct platform log structures were not always available, parallel school-based behavioral proxies (e.g., homework completion regularity and completion of scheduled learning tasks) were incorporated when feasible.

Mechanism-related variables were included to clarify how the intervention influenced student behavior. These variables included academic self-efficacy, school belongingness and perceived support, and academic emotion regulation indicators such as learning-related anxiety, frustration coping, and emotional recovery capacity. These measures were used to examine psychological pathways linking the intervention to SRL improvement and to explore whether the intervention supported motivational repair and emotional stabilization among disadvantaged underachieving students.

Secondary outcomes included broader academic and behavioral performance indicators, including academic performance or test scores, learning engagement, and persistence. Learning engagement was assessed across behavioral, cognitive, and emotional dimensions and, where feasible, was supplemented with behavioral evidence such as homework submission records and attendance or absence records to improve ecological validity. Persistence and learning-habit change were evaluated using indicators such as continued task completion, weekly goal attainment, review continuity, and sustained use of learning-support functions during the intervention and follow-up phases.

Process indicators were collected systematically to characterize intervention dosage and implementation quality, including interaction frequency, average interaction duration, weekly usage rates, module completion rates, strategy adoption rates, and review quality. Review quality was coded using structured criteria and supported by automated text-feature extraction to reflect the depth of reflection and self-monitoring. These process data were used for descriptive implementation analysis, adherence analysis, and dose–response exploration, and they also informed robustness checks regarding the source and stability of intervention effects. In all inferential models, potential confounding variables were prespecified and adjusted as appropriate, including baseline demographic characteristics, socioeconomic indicators, digital literacy and device access, and family learning support.

### 2.6 Statistical analysis

All primary and secondary analyses were conducted under an intention-to-treat framework to preserve the original cluster allocation and estimate the incremental effects of the intervention on post-intervention and follow-up outcomes. Because the trial used class-level randomization, all models explicitly accounted for clustering at the class level and repeated observations within students (Campbell et al., 2012). For continuous outcomes, linear mixed-effects models were fitted with fixed effects for group, time, and the group-by-time interaction, and with random effects specified to account for within-student repeated measures and class-level clustering. Baseline values of the outcome and prespecified covariates were included to improve precision and reduce bias from residual imbalance.

Because the primary outcome structure included both self-report SRL scales and behavioral SRL markers, the analytic strategy treated these as co-primary indicators of SRL change rather than classifying behavioral markers as process metrics only. Continuous behavioral variables were analyzed using mixed-effects models when distributions were approximately normal, whereas count or proportion outcomes (e.g., review frequency or completion rates) were analyzed using generalized mixed-effects models with appropriate link functions and distributional assumptions. This approach was used to strengthen inference regarding SRL change and reduce overreliance on self-reported improvement alone.

Mechanism testing was conducted using mediation analysis to evaluate indirect effects from intervention exposure to academic and engagement-related outcomes through changes in SRL. Indirect effects were estimated using bootstrap resampling with bias-corrected confidence intervals to improve robustness of inference (Hayes, 2018). Where model assumptions allowed, mediation models incorporated clustered standard errors or multilevel structures to remain consistent with the trial design. Equity-oriented heterogeneity analyses were conducted by including prespecified moderators (e.g., socioeconomic intensity, baseline underachievement severity, and digital literacy) and by conducting stratified analyses to identify differential effect patterns across subgroups.

Missing data were handled according to the assumed missing-data mechanism, primarily using maximum likelihood estimation within mixed models and multiple imputation as a complementary strategy when appropriate (Little and Rubin, 2019). Missingness patterns and attrition reasons were examined descriptively, and sensitivity analyses compared complete-case, imputed, and model-based estimates. Additional robustness analyses included adherence-adjusted analyses and alternative model specifications to evaluate the stability of effect estimates.

Because teacher and research-assistant oversight formed part of the intervention context, an exploratory analysis was prespecified to examine whether teacher check-in frequency or supervisor contact intensity was associated with changes in SRL and related learning outcomes. This analysis was included to clarify the potential contribution of human supportive presence alongside AI mentoring and to support a more cautious interpretation of intervention attribution.

## 3 Results

### 3.1 Baseline equivalence and sample description

A total of 168 students who met the predefined criteria for socioeconomic disadvantage and academic underachievement were enrolled and assigned at the class level to the intervention group (*n* = 84) or the control group (*n* = 84). The two groups were well balanced at baseline across demographic characteristics, including age, sex, and grade distribution. No systematic between-group differences were observed in contextual characteristics such as family structure or home language environment, supporting the adequacy of the class-level randomization procedure.

The groups were also comparable on socioeconomic indicators. Parental educational attainment, eligibility for school subsidy or meal-support programs, household learning-resource availability, and access to a quiet study space were similar between groups. Device access and internet connectivity were likewise balanced, with high rates of smartphone ownership in both groups. Digital literacy levels were generally moderate to low in both groups, which is consistent with the practical profile of socioeconomically disadvantaged students who have basic digital access but limited learning-oriented digital skills.

Baseline academic indicators were comparable across groups, including rank percentile, mathematics scores, language arts scores, homework submission rates, and recent attendance. No significant between-group differences were observed in overall self-regulated learning (SRL) or in SRL subdimensions, including goal setting, planning, self-monitoring, strategy use, and reflection. Baseline levels of academic self-efficacy and school belongingness were low in both groups, whereas learning-related anxiety and perceived stress were in the moderate range. As shown in Table 1, standardized mean differences were small across measured variables, and all between-group comparisons were non-significant, indicating adequate baseline equivalence for subsequent outcome analyses.

**Table 1:** Baseline characteristics and group equivalence.

Variable	Intervention (*n* = 84)	Control (*n* = 84)	Test/*p*-value	SMD
Age (years), mean ± SD	15.42 ± 0.86	15.38 ± 0.91	*t* = 0.28, *p* = 0.78	0.04
Female, *n* (%)	46 (54.8)	44 (52.4)	*χ*^2^ = 0.10, *p* = 0.75	0.05
Grade 10, *n* (%)	41 (48.8)	39 (46.4)	*χ*^2^ = 0.10, *p* = 0.75	0.05
Grade 11, *n* (%)	43 (51.2)	45 (53.6)	*χ*^2^ = 0.10, *p* = 0.75	0.05
Primary home language matches school language, *n* (%)	71 (84.5)	69 (82.1)	*χ*^2^ = 0.17, *p* = 0.68	0.06
Lives with both parents, *n* (%)	49 (58.3)	52 (61.9)	*χ*^2^ = 0.22, *p* = 0.64	0.07
Parent highest education ≤ high school, *n* (%)	56 (66.7)	53 (63.1)	*χ*^2^ = 0.24, *p* = 0.62	0.08
Eligible for school subsidy/free meal, *n* (%)	59 (70.2)	61 (72.6)	*χ*^2^ = 0.12, *p* = 0.73	0.05
Household learning resources index (0–10), mean ± SD	3.84 ± 1.57	3.96 ± 1.49	*t* = −0.50, *p* = 0.62	−0.08
Quiet study space at home (yes), *n* (%)	37 (44.0)	40 (47.6)	*χ*^2^ = 0.22, *p* = 0.64	−0.07
Owns a smartphone (yes), *n* (%)	76 (90.5)	75 (89.3)	*χ*^2^ = 0.05, *p* = 0.83	0.04
Reliable internet at home (yes), *n* (%)	62 (73.8)	64 (76.2)	*χ*^2^ = 0.12, *p* = 0.73	−0.06
Digital literacy (1–5), mean ± SD	2.91 ± 0.68	2.97 ± 0.72	*t* = −0.57, *p* = 0.57	−0.09
Baseline academic rank percentile (lower = worse), mean ± SD	24.6 ± 6.8	25.1 ± 7.2	*t* = −0.47, *p* = 0.64	−0.07
Math score (0–100), mean ± SD	56.7 ± 8.9	57.4 ± 9.1	*t* = −0.51, *p* = 0.61	−0.08
Language arts score (0–100), mean ± SD	58.9 ± 9.6	59.5 ± 9.3	*t* = −0.41, *p* = 0.68	−0.06
Homework submission rate (past 4 weeks, %), mean ± SD	63.4 ± 14.8	64.9 ± 15.2	*t* = −0.64, *p* = 0.52	−0.10
Attendance rate (past 4 weeks, %), mean ± SD	92.7 ± 5.9	93.1 ± 6.2	*t* = −0.43, *p* = 0.67	−0.06
SRL total (1–5), mean ± SD	2.36 ± 0.47	2.41 ± 0.49	*t* = −0.68, *p* = 0.50	−0.10
Goal setting (1–5), mean ± SD	2.28 ± 0.58	2.32 ± 0.61	*t* = −0.44, *p* = 0.66	−0.07
Planning (1–5), mean ± SD	2.21 ± 0.55	2.26 ± 0.57	*t* = −0.58, *p* = 0.56	−0.09
Self-monitoring (1–5), mean ± SD	2.33 ± 0.52	2.37 ± 0.54	*t* = −0.49, *p* = 0.62	−0.08
Strategy use (1–5), mean ± SD	2.42 ± 0.51	2.47 ± 0.53	*t* = −0.62, *p* = 0.53	−0.10
Reflection (1–5), mean ± SD	2.31 ± 0.56	2.35 ± 0.58	*t* = −0.46, *p* = 0.65	−0.07
Academic self-efficacy (1–5), mean ± SD	2.44 ± 0.63	2.48 ± 0.61	*t* = −0.42, *p* = 0.67	−0.06
School belonging (1–5), mean ± SD	2.68 ± 0.59	2.72 ± 0.57	*t* = −0.44, *p* = 0.66	−0.07
Learning anxiety (1–5), mean ± SD	3.12 ± 0.74	3.08 ± 0.77	*t* = 0.34, *p* = 0.73	0.05
Perceived stress (0–40), mean ± SD	19.6 ± 6.1	19.1 ± 6.4	*t* = 0.52, *p* = 0.60	0.08
Growth mindset (1–6), mean ± SD	3.71 ± 0.82	3.78 ± 0.79	*t* = −0.58, *p* = 0.56	−0.09
Teacher-rated learning engagement (1–5), mean ± SD	2.39 ± 0.66	2.43 ± 0.64	*t* = −0.40, *p* = 0.69	−0.06
Prior use of any learning app (yes), *n* (%)	29 (34.5)	31 (36.9)	*χ*^2^ = 0.11, *p* = 0.74	−0.05

### 3.2 Intervention engagement and implementation fidelity

Intervention engagement and implementation fidelity were evaluated using platform exposure, completion of core SRL workflow components, tiered support uptake, protocol adherence, and safety-governance indicators. Overall, students in the intervention group showed high initial uptake and sustained participation across the 8-week intervention period. Account activation within Week 1 was high, and participants maintained stable login frequency and repeated mentor-chat engagement over time. In contrast, the control group showed limited use of platform-based learning resources, which was consistent with the intended minimal-exposure design of the control condition. Detailed exposure and implementation metrics are presented in Table 2.

**Table 2:** Intervention exposure and implementation fidelity metrics.

Metric	Intervention (*n* = 84)	Control (*n* = 84)
Exposure and dose		
Activated account within Week 1, *n* (%)	81 (96.4)	83 (98.8)
Total platform logins (8 weeks), mean ± SD	31.7 ± 12.4	8.6 ± 5.1
Active weeks (≥1 login/week), mean ± SD	6.6 ± 1.7	2.3 ± 1.4
Total mentor-chat sessions, mean ± SD	18.9 ± 7.6	0.0 ± 0.0
Sessions per week, mean ± SD	2.36 ± 0.95	0.00 ± 0.00
Student messages sent (count), mean ± SD	146.3 ± 68.7	4.9 ± 6.8
AI mentor messages delivered (count), mean ± SD	212.4 ± 94.1	0.0 ± 0.0
Total time in mentor-chat (minutes), mean ± SD	126.8 ± 61.5	9.7 ± 12.9
Median session length (minutes), median [IQR]	6.1 [4.4, 8.7]	2.1 [1.3, 3.5]
Core SRL workflow completion		
Weekly structured check-ins completed (0–8), mean ± SD	6.9 ± 1.6	0.8 ± 1.1
Weekly goals created (0–8), mean ± SD	7.2 ± 1.3	1.1 ± 1.4
Action plans generated (0–8), mean ± SD	6.8 ± 1.7	0.9 ± 1.2
On-time weekly plan submission (%, per student), mean ± SD	74.5 ± 18.9	21.4 ± 17.6
Micro-tasks completed (0–40), mean ± SD	26.4 ± 9.8	9.2 ± 7.7
Daily reminder response rate (%), mean ± SD	63.1 ± 20.7	28.6 ± 19.3
Tiered support uptake and quality		
Tier-1 prompts delivered (count), mean ± SD	58.6 ± 14.9	12.3 ± 9.4
Tier-1 completion (completed/assigned, %), mean ± SD	78.2 ± 16.4	34.1 ± 20.5
Tier-2 reflective sessions completed (0–6), mean ± SD	4.7 ± 1.9	0.6 ± 0.9
Reflection submissions (count), mean ± SD	6.2 ± 2.7	0.7 ± 1.0
Reflection depth score (1–5), mean ± SD	3.24 ± 0.71	2.18 ± 0.64
Tier-3 adaptive challenges attempted (0–3), mean ± SD	2.1 ± 0.9	0.2 ± 0.5
Mastery-oriented feedback delivered (count), mean ± SD	11.6 ± 5.8	1.9 ± 2.7
Strategy selection events (count), mean ± SD	22.7 ± 9.4	3.6 ± 4.8
Strategy adoption rate (self-reported used/selected, %), mean ± SD	68.4 ± 17.2	33.8 ± 18.6
Retention and protocol adherence		
Completed ≥6 of 8 core weeks, *n* (%)	72 (85.7)	38 (45.2)
Completed post-test (T1), *n* (%)	80 (95.2)	79 (94.0)
Completed follow-up (T2), *n* (%)	78 (92.9)	76 (90.5)
Discontinued intervention early, *n* (%)	6 (7.1)	3 (3.6)
Safety and human oversight		
Automated risk flags triggered (count), mean ± SD	0.86 ± 1.12	0.21 ± 0.54
Students with ≥1 risk flag, *n* (%)	24 (28.6)	9 (10.7)
Human check-ins initiated (count), mean ± SD	0.31 ± 0.61	0.08 ± 0.30
Escalations to school support staff, *n* (%)	4 (4.8)	1 (1.2)
Adverse events judged study-related, *n* (%)	0 (0.0)	0 (0.0)

Completion rates for the core SRL workflow were high in the intervention group. Students completed most weekly structured check-ins, created weekly goals regularly, and generated action plans at a high rate. On-time plan submission was also substantially higher in the intervention group than in the control group. In addition, intervention participants completed more micro-tasks and responded more frequently to daily reminders, indicating that the AI-supported format was feasible under the practical constraints faced by this student population. These indicators provide behavioral evidence that participants engaged not only with the platform itself, but also with the intended planning and self-management routines embedded in the intervention.

Process indicators related to reflection and strategic adjustment also showed sustained engagement with the reflective components of the intervention. The intervention group completed substantially more reflective sessions and submitted more review entries than the control group, and reflection depth scores were higher in the intervention group. Together, these results indicate that students engaged with reflective review and attribution-focused monitoring rather than limiting participation to basic check-ins or task completion alone. This pattern is consistent with the intervention design, which emphasized progressive strengthening of monitoring and reflection across the SRL cycle.

Uptake of the tiered support structure was likewise strong. Delivery and completion rates for foundational prompts were high, reflective-session completion was sustained, and a substantial proportion of students progressed to the adaptive challenge modules in the later phase. Indicators of strategy selection and strategy adoption further suggest that students actively engaged with strategy-support components rather than passively receiving messages. This is important for interpreting outcome changes because the intervention was designed to strengthen strategic autonomy and perceived control, rather than simply increase contact frequency.

Protocol adherence remained high through both post-intervention and follow-up assessments. In the intervention group, most participants completed at least six of the eight core intervention weeks, and post-test and follow-up completion rates were high in both groups. Early discontinuation was limited. These patterns support the feasibility of implementing the program in real school settings and reduce concern that observed effects were driven primarily by selective retention.

Safety and oversight indicators further showed that the governance framework functioned as intended. Automated risk flags were more frequent in the intervention group, which is expected given the greater volume of monitored interactions and the higher likelihood of detecting distress signals in a structured mentoring environment. Human check-ins and escalations to school support staff were documented when needed, and no study-related adverse events were identified. These findings support the practical viability of combining AI-supported mentoring with human-supervised safety procedures in school-based implementation.

Exploratory analysis of human oversight intensity showed that teacher check-in frequency and supervisor contact intensity were not independently associated with changes in SRL after adjustment for baseline SRL, group allocation, and platform engagement indicators. Inclusion of oversight-related variables in the mixed-effects models did not materially alter the estimated intervention effects on SRL total, SRL subdimensions, or behavioral co-primary SRL markers. These findings suggest that the observed gains were not explained solely by variation in human supportive contact, although the intervention should be interpreted as an AI-supported model implemented within a supervised school context.

### 3.3 Concept-to-system translation of the intervention

To examine whether the proposed intervention was not only theoretically grounded but also operationally implementable, results are presented at both the conceptual and system levels. The findings indicate that the intervention functioned as more than an abstract self-regulation framework. It was implemented as an executable, supervisable, and trackable closed-loop support system within authentic school settings. At the conceptual level, the intervention specified trainable components and cyclical SRL processes across planning, execution, and reflection. At the system level, these components were translated into interacting platform modules with corresponding logs and governance mechanisms, allowing intervention dosage, implementation quality, and safety boundaries to be monitored throughout the study. The operational translation of these SRL components into platform-guided mentoring interactions was further supported by the version-controlled prompt framework and representative templates provided in Appendix A.

**Figure 1:**
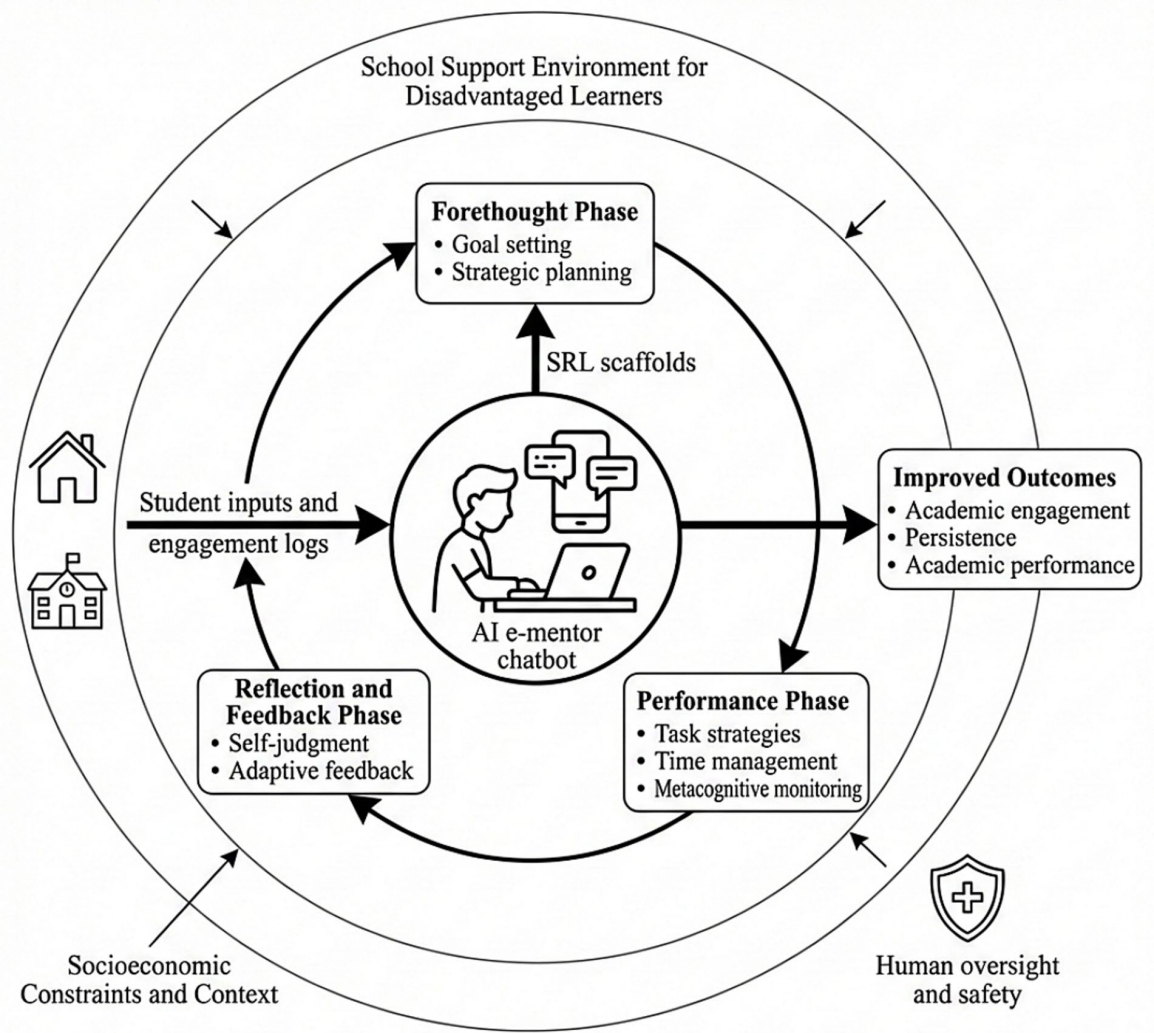
AI-supported e-mentoring concept for improving self-regulated learning among socioeconomically disadvantaged underachieving students.

Figure 1 shows how the intervention pathway was mapped onto the classic three-stage self-regulated learning cycle, including forethought (planning), performance (execution), and self-reflection (regulation and feedback). In the forethought phase, the intervention emphasized goal clarification and planning to support learning initiation. During the performance phase, the intervention supported continuity through strategy prompts, time-management routines, and metacognitive monitoring. In the reflective phase, the system supported strategy calibration and motivational adjustment through self-evaluation and adaptive feedback. This framework highlights that the core difficulties of socioeconomically disadvantaged underachieving students are not limited to knowledge deficits, but also involve disruptions in learning initiation, continuity of execution, and reflective consolidation. Accordingly, AI-supported e-mentoring was designed as a continuous scaffold rather than a one-time support event, enabling abstract regulatory processes to be externalized into manageable action units and reducing the psychological and execution burden associated with sustained self-regulation.

**Figure 2:**
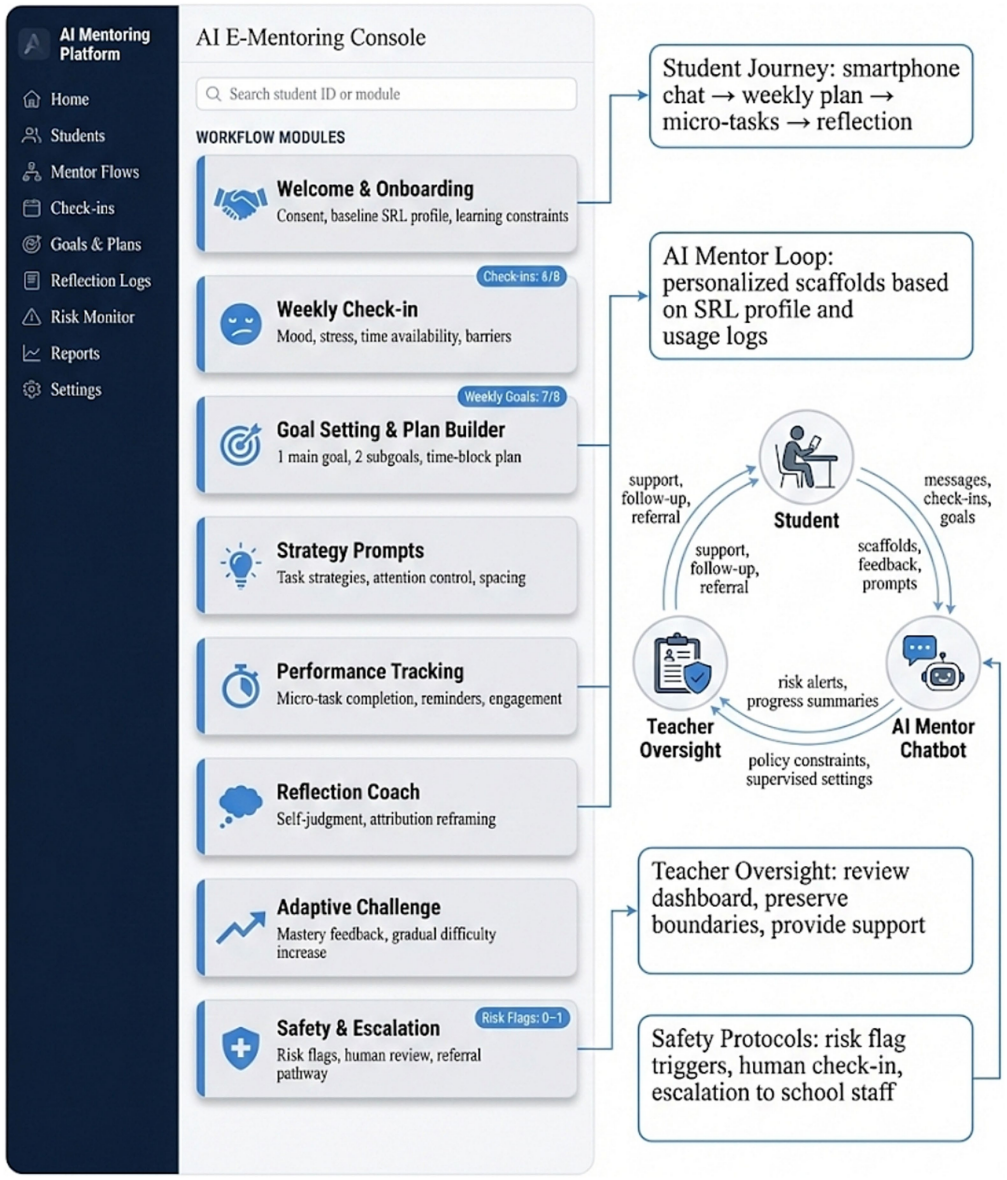
System architecture and user journey of the AI-supported e-mentoring platform (student–AI mentor–teacher oversight loop).

Figure 2 presents the closed-loop support architecture comprising three interrelated components: student-side interaction, AI mentor guidance, and school-side oversight. On the student side, weekly learning cycles were completed through structured check-ins, goal setting, plan generation, micro-task completion, and reflective review. These process data were recorded in real time as indicators of behavioral engagement and participation, thereby providing objective support for later effectiveness and mechanism analyses. On the AI mentor side, tiered support was generated using baseline SRL profiles and ongoing process logs, ranging from foundational goal-setting scaffolds to more intensive reflective guidance and adaptive challenges, which supported progression from initiation to consolidation. On the school side, teachers and designated supervisors did not replace the mentoring process but provided boundary governance and risk oversight, including progress review and alert response when needed. This arrangement preserved the intervention’s mentoring structure while strengthening safety assurance and ethical compliance for vulnerable adolescent participants.

### 3.4 Primary effects on self-regulated learning

The primary outcome was change in self-regulated learning (SRL) over time and between-group differences in SRL levels. Across the intervention period, students in the intervention group showed clear improvement in total SRL and in all measured SRL subdimensions at post-intervention, whereas the control group showed only modest increases or small fluctuations. These group differences remained evident at follow-up. Adjusted linear mixed-effects models showed statistically significant between-group advantages for the intervention condition at both post-intervention (T1) and follow-up (T2), indicating that AI-supported e-mentoring combined with structured SRL scaffolding produced incremental improvements in SRL under real school conditions. Detailed estimates are shown in Table 3.

**Table 3:** Primary outcomes: effects on self-regulated learning over time.

Time point/estimate	SRL total	Goal setting	Planning	Self-monitoring	Strategy use	Reflection
T0 Baseline, Intervention (*n* = 84)	2.36 ± 0.47	2.28 ± 0.58	2.21 ± 0.55	2.33 ± 0.52	2.42 ± 0.51	2.31 ± 0.56
T0 Baseline, Control (*n* = 84)	2.41 ± 0.49	2.32 ± 0.61	2.26 ± 0.57	2.37 ± 0.54	2.47 ± 0.53	2.35 ± 0.58
T1 Post, Intervention (*n* = 80)	3.05 ± 0.56	3.12 ± 0.64	3.02 ± 0.61	2.97 ± 0.58	3.06 ± 0.57	3.08 ± 0.63
T1 Post, Control (*n* = 79)	2.58 ± 0.53	2.63 ± 0.60	2.52 ± 0.58	2.54 ± 0.55	2.63 ± 0.54	2.58 ± 0.60
T1 Adjusted group difference (*β*, 95% CI); *p*-value	0.41 (0.28, 0.54); *p* < 0.001	0.44 (0.28, 0.60); *p* < 0.001	0.45 (0.30, 0.60); *p* < 0.001	0.38 (0.24, 0.52); *p* < 0.001	0.37 (0.23, 0.51); *p* < 0.001	0.44 (0.29, 0.60); *p* < 0.001
T2 Follow-up, Intervention (*n* = 78)	2.95 ± 0.55	3.01 ± 0.63	2.92 ± 0.60	2.88 ± 0.57	2.98 ± 0.56	2.97 ± 0.61
T2 Follow-up, Control (*n* = 76)	2.55 ± 0.52	2.57 ± 0.59	2.48 ± 0.57	2.51 ± 0.54	2.60 ± 0.53	2.53 ± 0.58
T2 Adjusted group difference (*β*, 95% CI); *p*-value	0.36 (0.22, 0.49); *p* < 0.001	0.38 (0.22, 0.54); *p* < 0.001	0.39 (0.23, 0.55); *p* < 0.001	0.33 (0.19, 0.47); *p* < 0.001	0.33 (0.19, 0.47); *p* < 0.001	0.39 (0.24, 0.55); *p* < 0.001

The pattern of effects across SRL dimensions was also consistent with the intervention design. The largest adjusted between-group differences were observed in goal setting, planning, and reflection at T1, with similarly strong effects retained at T2. This pattern aligns with the intervention’s emphasis on low-threshold task initiation, structured planning, and guided review. Improvements were also observed in self-monitoring and strategy use, suggesting that intervention effects extended beyond early-stage planning to the execution phase of the SRL cycle. In other words, students did not only become better at setting goals; they also demonstrated stronger ongoing regulation during task performance.

Follow-up results suggested partial attenuation after the end of the intensive intervention phase, but the intervention advantage remained statistically and practically meaningful. For example, the adjusted between-group difference in SRL total decreased from *β* = 0.41 at T1 to *β* = 0.36 at T2, while remaining significant. A similar pattern was observed across SRL subdimensions. These results indicate that some tapering occurred after active support was reduced, but the overall pattern remained consistent with short-term maintenance of gains rather than immediate regression to baseline. This finding is important because it suggests that the intervention supported changes in SRL routines that persisted beyond the active implementation window, even if some weakening in effect magnitude was observed.

Table 3 further shows that the intervention group improved from low baseline SRL levels to clearly higher post-intervention scores across all dimensions, while the control group remained closer to baseline levels. This pattern is consistent with the intervention’s intended role as a scaffold for reconstructing the SRL cycle in students who initially showed weak planning, monitoring, and reflective capacity.

#### 3.4.1 Co-primary behavioral SRL outcomes

In addition to scale-based SRL outcomes, behavioral SRL markers were analyzed as co-primary outcomes to reduce overreliance on self-report improvement and to provide observable evidence of self-regulatory behavior change. Although several of these indicators are also reported descriptively in Table 2 as implementation metrics, Table 4 presents them as co-primary behavioral SRL outcomes using adjusted inferential models consistent with the prespecified analytic framework.

**Table 4:** Co-primary behavioral SRL outcomes during the intervention period.

Behavioral SRL marker	Intervention *n* = 84	Control *n* = 84	Adjusted group difference at T1 (95% CI); *p*-value
Weekly structured check-ins completed (0–8)	6.9 ± 1.6	0.8 ± 1.1	5.84 (5.34, 6.34); *p* < 0.001
On-time weekly plan submission (% per student)	74.5 ± 18.9	21.4 ± 17.6	50.92 (45.11, 56.73); *p* < 0.001
Weekly goal attainment rate (% per student)	68.7 ± 17.4	39.6 ± 16.8	27.84 (22.36, 33.32); *p* < 0.001
Micro-tasks completed (0–40)	26.4 ± 9.8	9.2 ± 7.7	15.76 (13.01, 18.51); *p* < 0.001
Review submissions (count, 8 weeks)	6.2 ± 2.7	0.7 ± 1.0	5.18 (4.46, 5.90); *p* < 0.001
Students with consistent review continuity (≥4 reviews), *n* (%)	61 (72.6)	8 (9.5)	OR 24.13 (10.21, 57.03); *p* < 0.001
Students achieving ≥70% on-time plan submission, *n* (%)	49 (58.3)	7 (8.3)	OR 15.92 (6.59, 38.46); *p* < 0.001

Across the intervention period, students in the intervention group demonstrated significantly stronger performance on planning-related and self-monitoring-related behavioral indicators than students in the control group. These differences were observed in on-time weekly plan submission, weekly goal attainment, micro-task completion, review submission frequency, and weekly structured check-in completion. Adjusted mixed-effects and generalized mixed-effects models showed consistent between-group advantages for the intervention condition across these behavioral SRL markers (Table 4).

The pattern of effects was aligned with the intervention’s mechanism design. Indicators linked to planning initiation and routine formation (e.g., weekly plan submission and structured check-ins) showed the largest between-group differences, while indicators reflecting execution and reflective continuity (e.g., micro-task completion and review submission) also showed robust improvements. Together with the scale-based results in Table 3, these findings strengthen the interpretation that the intervention improved not only perceived SRL capacity but also enacted self-regulatory routines in daily learning practice. The convergence of self-report and behavioral indicators reduces the likelihood that the observed SRL gains can be explained solely by social desirability or response-shift effects.

**Figure 3:**
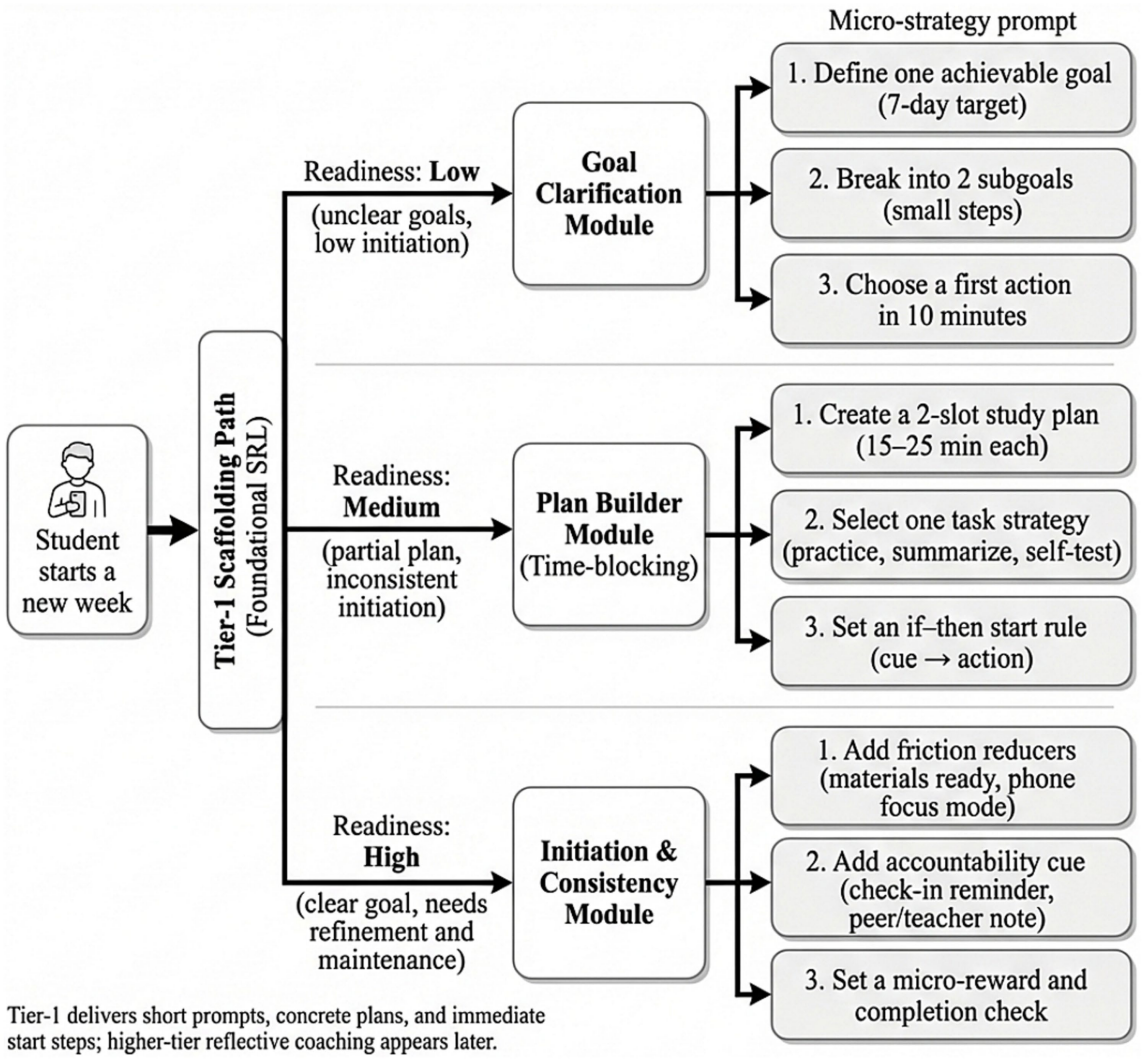
Tier-1 support: Goal clarification and micro-strategy prompts for foundational self-regulation (planning and task initiation).

Evidence for mechanism consistency is further supported by the correspondence between intervention content and the observed pattern of primary outcomes. As illustrated in Figure 3, Tier-1 support focused on common bottlenecks in disadvantaged underachieving students, particularly goal ambiguity and difficulty initiating study behavior. Through hierarchical prompts, the system translated key SRL elements such as goal clarification, task decomposition, and immediate initiation into operational micro-strategy guidance. This structure mirrors the strongest early gains observed in planning-related SRL dimensions and supports coherence between the conceptual framework and system-level intervention design.

**Figure 4:**
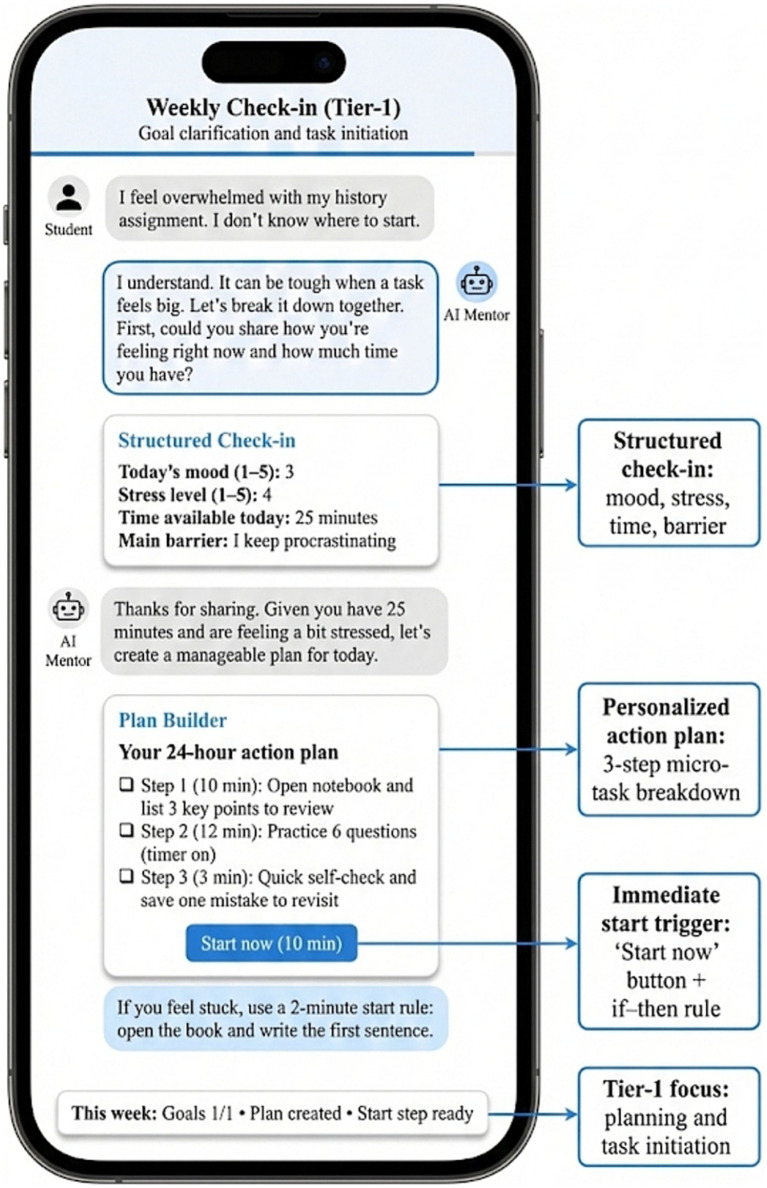
Tier-1 interaction interface: structured check-in and personalized action-plan screen.

Figure 4 shows the structure of the check-in and personalized action-plan interface, which integrated emotion and stress identification, time-availability assessment, and barrier recognition in a single interaction sequence. Within this interface, students generated a three-step action plan supported by selectable checkpoints and an immediate-start function. This closed-loop interaction translated self-regulation from an abstract psychological construct into a sequence of actionable choices in real time. The interface-level design is consistent with the observed improvement in execution-related SRL indicators and provides descriptive support for alignment between system functions and user behavior.

### 3.5 Secondary effects on academic performance, engagement, and persistence

Secondary analyses examined academic performance, learning engagement, and persistence to determine whether improvements in SRL were accompanied by broader and educationally meaningful changes. Across these domains, the intervention group showed more favorable outcomes than the control group at post-intervention. These findings indicate that the AI-supported e-mentoring program was associated not only with improvement in self-regulated learning, but also with change in behavioral indicators and academic outcomes that are directly relevant to school functioning. Detailed estimates are shown in Table 5.

**Table 5:** Secondary outcomes: academic performance, engagement, and persistence.

Outcome	Intervention T0 (*n* = 84)	Control T0 (*n* = 84)	Intervention T1 (*n* = 80)	Control T1 (*n* = 79)	Adjusted Δ at T1 (95% CI); *p*
Academic performance					
Composite grade average (0–100)	57.8 ± 8.1	58.2 ± 8.3	64.2 ± 8.7	60.8 ± 8.5	3.05 (1.39, 4.71); *p* < 0.001
Math score (0–100)	56.7 ± 8.9	57.4 ± 9.1	63.9 ± 9.4	59.6 ± 9.2	3.72 (1.84, 5.60); *p* < 0.001
Language arts score (0–100)	58.9 ± 9.6	59.5 ± 9.3	64.8 ± 9.7	61.6 ± 9.4	2.86 (0.98, 4.74); *p* = 0.003
Engagement					
Homework submission rate (%)	63.4 ± 14.8	64.9 ± 15.2	77.3 ± 13.6	68.1 ± 14.7	8.18 (4.32, 12.04); *p* < 0.001
Teacher-rated engagement (1–5)	2.39 ± 0.66	2.43 ± 0.64	3.02 ± 0.69	2.62 ± 0.66	0.36 (0.18, 0.54); *p* < 0.001
Attendance rate (%)	92.7 ± 5.9	93.1 ± 6.2	95.6 ± 4.8	93.7 ± 5.7	1.62 (0.28, 2.95); *p* = 0.018
Persistence					
Study persistence scale (1–5)	2.46 ± 0.62	2.50 ± 0.60	3.12 ± 0.66	2.71 ± 0.63	0.37 (0.19, 0.55); *p* < 0.001
Consistent engagement (≥6 of 8 weeks), *n* (%)	35 (41.7)	33 (39.3)	72 (85.7)	38 (48.1)	OR 6.21 (3.12, 12.36); *p* < 0.001

In the academic domain, students in the intervention group showed significantly larger improvements in composite grade average and in both mathematics and language arts scores than students in the control group. This pattern is consistent with the interpretation that stronger alignment between goal setting, planning, and execution led to more effective use of available study time. By contrast, changes in the control group were smaller and more consistent with expected short-term variation in routine school performance. These results support the broader argument that underachievement in this population often reflects disruption in learning processes and self-regulation rather than fixed limitations in learning potential.

In the engagement domain, the intervention group showed larger improvements in homework submission rates, teacher-rated engagement, and attendance. These changes suggest that the intervention influenced not only task completion frequency but also the continuity and quality of student participation in everyday learning. The observed engagement gains were consistent with a pattern of more stable participation in manageable learning steps, rather than sporadic engagement with overly demanding tasks. This is consistent with the intervention’s modular task design and repeated prompts for low-threshold action initiation.

Persistence-related outcomes showed a similarly strong pattern. The intervention group demonstrated significantly higher post-intervention persistence scores and substantially higher rates of consistent engagement across the 8-week period. Under comparable academic demands and family resource constraints, this pattern suggests that the intervention reduced disengagement and supported sustained participation. The large between-group difference in consistent engagement is especially important because it reflects a behavioral pattern that is difficult to detect using self-report measures alone and aligns closely with the intervention’s intended function as a regulatory scaffold.

**Figure 5:**
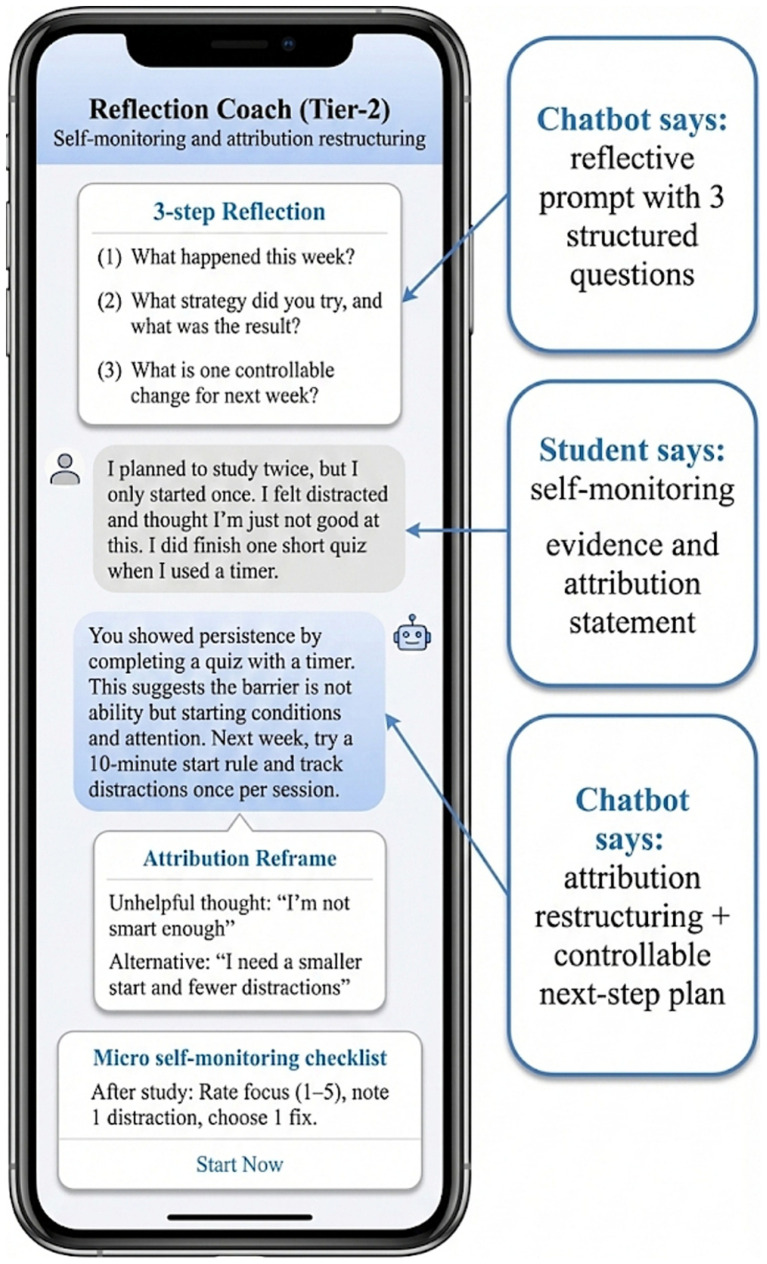
Tier-2 support: reflective coaching for self-monitoring and attributional restructuring.

The secondary outcome pattern was also consistent with the intervention’s reflective and attributional components. As shown conceptually in Figure 5, the Tier-2 reflective coaching module used students’ own process data to guide structured reflection on controllable factors affecting learning outcomes. The goal of this module was to shift interpretation away from rigid, ability-based explanations and toward modifiable strategies and contextual adjustment. The observed improvements in persistence and engagement, together with the primary SRL findings, are consistent with this mechanism pathway.

**Figure 6:**
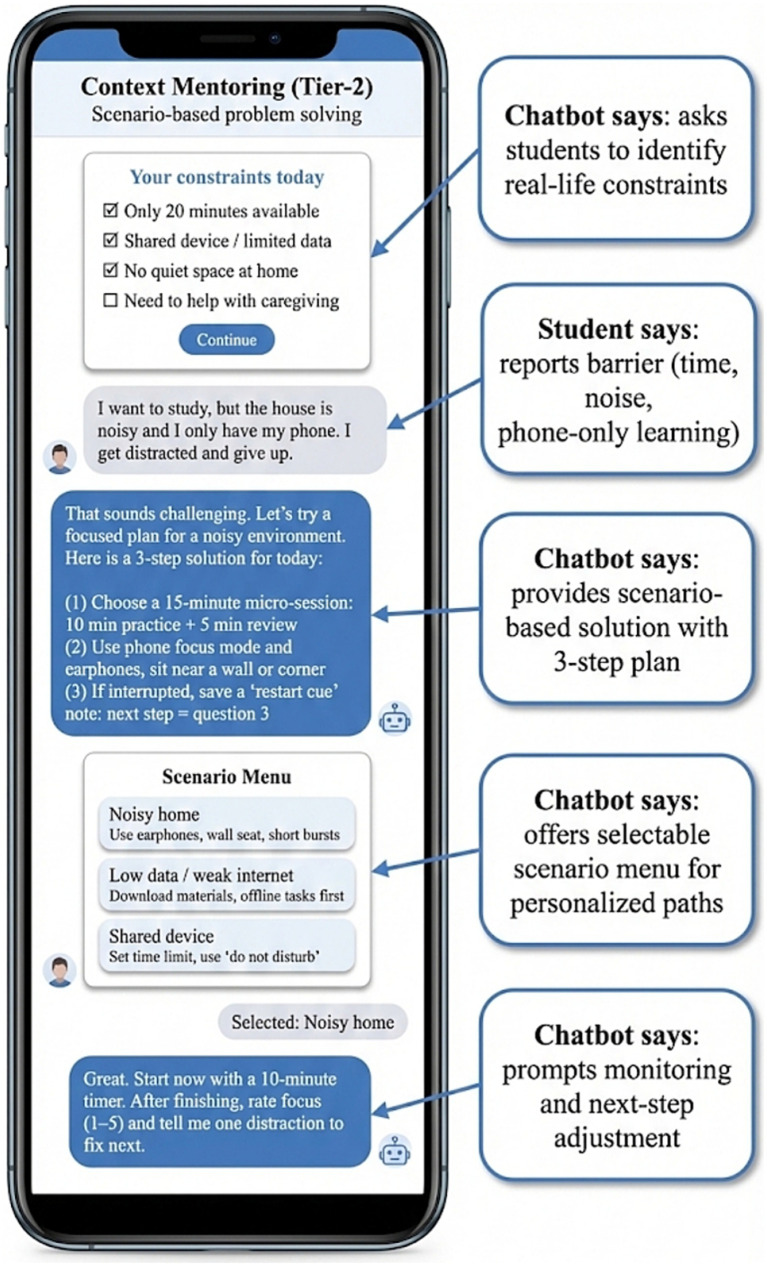
Tier-2 contextualized mentoring: scenario-based problem solving aligned with students’ real-life constraints.

Figure 6 illustrates the contextualized mentoring design used to incorporate real-life constraints into ongoing learning decisions. The system accounted for fragmented time, household disruptions, limited device access, and caregiving or family responsibilities that often shape disadvantaged students’ learning environments. By incorporating these constraints into plan generation and restart cues, the system supported feasible re-engagement after interruption. This design feature is consistent with the observed improvements in persistence and engagement, particularly in students who might otherwise disengage after disrupted study attempts.

### 3.6 Mechanism, equity, and robustness analyses

To clarify how the intervention functioned, which students benefited most, and whether the findings were sensitive to modeling assumptions or missing-data handling strategies, additional analyses were conducted beyond the primary effect models. These analyses included mediation tests, moderation and equity analyses, and robustness and sensitivity checks. Across these analyses, a consistent pattern emerged. AI-supported e-mentoring was associated with improvements in self-regulated learning (SRL), and these SRL gains were in turn associated with improvements in academic and behavioral outcomes. In addition, intervention benefits tended to be larger among students with lower baseline resources and higher initial learning risk. The direction and magnitude of these patterns remained broadly stable across alternative specifications.

Mechanism analyses indicated partial mediation of intervention effects through SRL. Specifically, the intervention was associated with substantial improvement in SRL, and higher post-intervention SRL was significantly associated with better composite grades, higher homework submission rates, stronger teacher-rated engagement, and greater study persistence. When SRL was entered into the outcome models, the direct intervention effects were attenuated, but bootstrap-based indirect effects remained statistically significant across outcomes. This pattern supports the interpretation that SRL functioned as a proximal mechanism linking intervention exposure to broader learning-related outcomes. As shown in Table 6, the estimated proportion mediated was substantial across domains, with indirect effects accounting for a large share of the total intervention effect. These findings are consistent with the intervention’s theoretical premise that, for socioeconomically disadvantaged underachieving students, constraints often arise from breakdowns in learning initiation, process regulation, and post-difficulty recovery rather than from lack of learning content alone.

**Table 6:** Mediation models: intervention → self-regulated learning → outcomes (T1).

Outcome (T1)	a-path: Intervention → SRL (β, 95% CI)	b-path: SRL → Outcome (β, 95% CI)	Total effect c (β, 95% CI)	Direct effect c′ (β, 95% CI)	Indirect effect a × b (boot 95% CI)	*p* (indirect)	% mediated
Composite grade average (0–100)	0.41 (0.28, 0.54)	5.38 (3.72, 7.09)	3.05 (1.39, 4.71)	0.86 (0.09, 1.63)	2.21 (1.33, 3.23)	<0.001	72.5
Homework submission rate (%)	0.41 (0.28, 0.54)	14.6 (10.2, 19.4)	8.18 (4.32, 12.04)	2.18 (0.52, 3.86)	5.98 (3.62, 8.61)	<0.001	73.1
Study persistence scale (1–5)	0.41 (0.28, 0.54)	0.62 (0.45, 0.79)	0.37 (0.19, 0.55)	0.12 (0.01, 0.23)	0.25 (0.15, 0.36)	<0.001	67.6
Teacher-rated engagement (1–5)	0.41 (0.28, 0.54)	0.55 (0.39, 0.71)	0.36 (0.18, 0.54)	0.13 (0.03, 0.24)	0.23 (0.14, 0.33)	<0.001	63.9

Moderation and equity analyses further showed that intervention effects were not evenly distributed across all students. Larger effects on SRL at T1 were observed among students with higher socioeconomic disadvantage, lower baseline SRL, and greater prior underachievement severity. In contrast, no clear interaction effects were observed for gender or grade level. The interaction pattern for digital access constraint was also notable. Rather than weakening the intervention, limited or shared device/data access was associated with a modestly stronger intervention effect, which is consistent with the design emphasis on low-threshold, context-adapted support for students facing constrained learning conditions. Overall, these moderation results suggest that the intervention operated in a compensatory direction, with relatively greater benefits among students with higher baseline vulnerability. Detailed interaction estimates are reported in Table 7.

**Table 7:** Moderation and equity analysis: differential intervention effects across baseline risk profiles.

Moderator (baseline)	Levels/coding	SRL T1 main effect (β)	Interaction: Intervention × Moderator (β, 95% CI)	*p* (interaction)
Socioeconomic disadvantage index (z)	Higher = more disadvantage	0.41	0.11 (0.03, 0.19)	0.008
Baseline SRL Total (1–5)	Centered	0.41	−0.18 (−0.27, −0.08)	<0.001
Prior underachievement severity (z)	Lower prior grades = higher severity	0.41	0.09 (0.01, 0.17)	0.028
Digital access constraint (0/1)	1 = shared/limited device or data	0.41	0.07 (0.00, 0.14)	0.046
Gender (0/1)	1 = female	0.41	0.03 (−0.06, 0.12)	0.52
Grade level (0/1)	1 = higher grade within sample	0.41	−0.05 (−0.13, 0.04)	0.27

Robustness and sensitivity analyses showed that the primary conclusions were not driven by a single analytic choice. Across alternative model specifications, including school fixed effects (where applicable), cluster-robust standard errors, per-protocol analyses, multiple imputation for missing data, inverse probability weighting for attrition, and exclusion of exposure outliers, the intervention effects on SRL, composite grades, and homework submission remained directionally consistent and statistically significant. Although point estimates varied modestly across specifications, the overall pattern of effects was stable and remained within a reasonable range. These findings reduce concern that the main conclusions depend on a narrow set of statistical assumptions.

Missing-data diagnostics also supported the stability of the results. Baseline predictors did not show significant associations with T1 missingness in the diagnostic models summarized in Table 8, suggesting a low likelihood of systematic differential attrition by group assignment or baseline risk profile. Taken together, the robustness and missingness analyses support the internal consistency of the findings and strengthen confidence in the intervention effect estimates.

**Table 8:** Robustness, sensitivity, and missing-data diagnostics.

Analysis/specification	SRL total effect at T1 (β, 95% CI)	*p*-value	Composite grade effect at T1 (β, 95% CI)	*p*-value	Homework submission effect at T1 (β, 95% CI)	*p*-value
Primary ITT mixed-effects (baseline-adjusted; class cluster)	0.41 (0.28, 0.54)	<0.001	3.05 (1.39, 4.71)	<0.001	8.18 (4.32, 12.04)	<0.001
Add school fixed effects (if multi-school)	0.39 (0.26, 0.52)	<0.001	2.89 (1.21, 4.57)	0.001	7.74 (3.91, 11.58)	<0.001
Cluster-robust SE (HC3)	0.40 (0.27, 0.53)	<0.001	3.02 (1.33, 4.70)	<0.001	8.05 (4.01, 12.09)	<0.001
Per-protocol (≥6/8 weeks engaged)	0.47 (0.33, 0.61)	<0.001	3.68 (1.86, 5.50)	<0.001	9.46 (5.22, 13.71)	<0.001
Multiple imputation (MICE, *m* = 30) for missing T1	0.40 (0.27, 0.53)	<0.001	2.97 (1.30, 4.64)	<0.001	7.93 (4.03, 11.83)	<0.001
Inverse probability weighting (attrition-adjusted)	0.38 (0.24, 0.52)	<0.001	2.76 (1.01, 4.51)	0.002	7.31 (3.12, 11.50)	0.001
Sensitivity: exclude top 5% exposure outliers	0.39 (0.26, 0.52)	<0.001	2.84 (1.18, 4.50)	0.001	7.62 (3.68, 11.56)	<0.001
Missingness diagnostic: baseline predictors of T1 missingness (logit)	OR 1.06 (0.86, 1.31)	0.58	OR 0.97 (0.79, 1.20)	0.78	OR 1.09 (0.88, 1.34)	0.43

**Figure 7:**
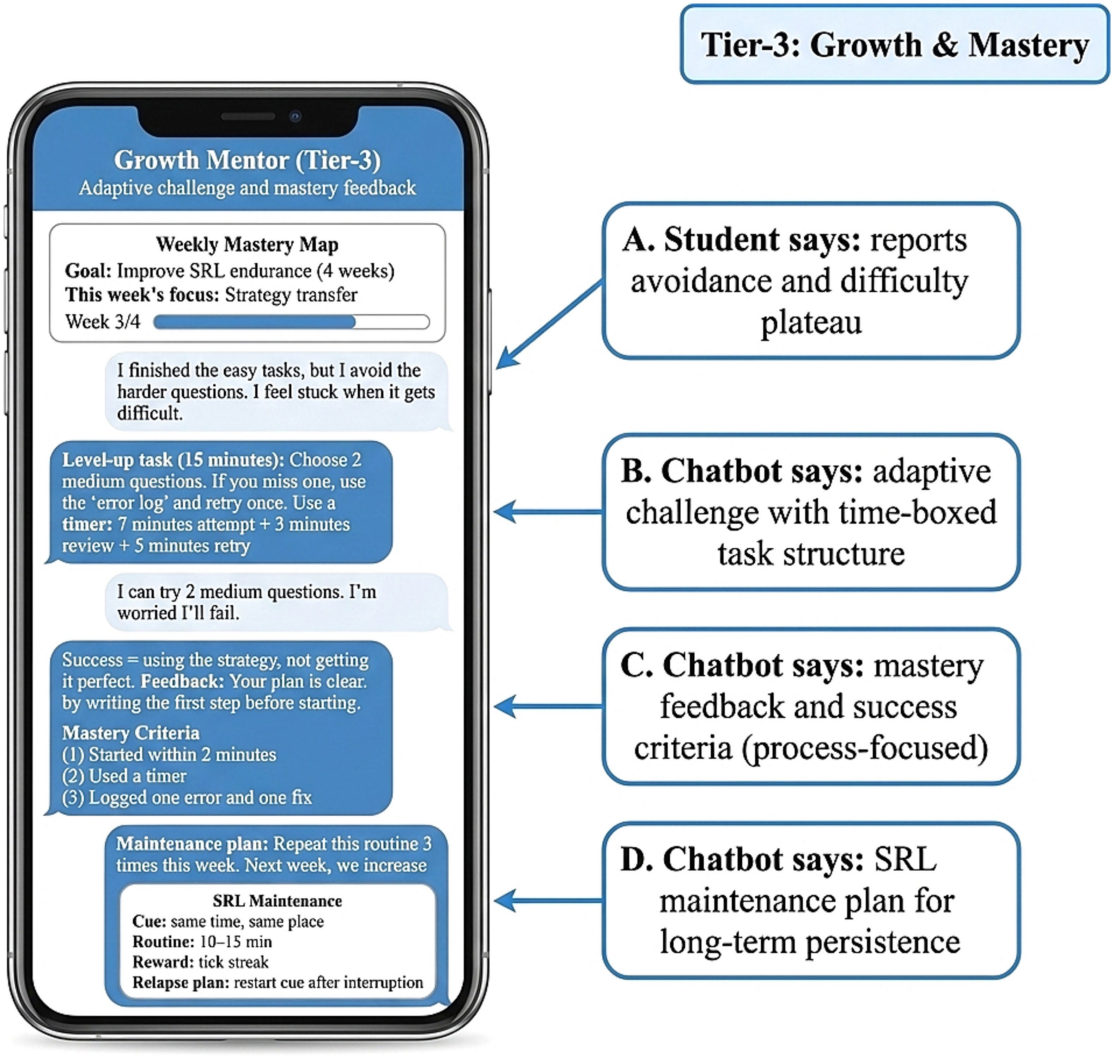
Tier-3 growth-oriented mentoring: adaptive challenge, mastery feedback, and longer-term SRL maintenance.

To further characterize mechanisms related to maintenance of effects, additional results examined the function of the Tier-3 growth-oriented mentoring module. As shown in Figure 7, Tier-3 support emphasized adaptive challenge, mastery criteria, and maintenance planning rather than immediate error correction. The module was designed to shift students’ learning orientation from short-term outcome completion toward process-based mastery and continuity. By gradually increasing task difficulty, defining mastery targets, and incorporating restart prompts and continuity goals after interruption, the module reduced the likelihood of disengagement during difficult periods. This pattern is consistent with the broader mechanism findings and provides descriptive support for the interpretation that SRL functioned not only as a mediator of short-term gains but also as a sustaining process that supported partial maintenance of effects beyond the intensive intervention phase.

## 4 Discussion

This study focused on socioeconomically disadvantaged students with persistent academic underachievement and examined whether AI-supported e-mentoring combined with structured self-regulated learning (SRL) training could improve both learning processes and observable educational outcomes. The findings indicate that the intervention was associated with meaningful improvements in core SRL dimensions, including goal setting, planning, self-monitoring, and reflection, together with gains in academic performance, learning engagement, and persistence (Mercier et al., 2024). This pattern supports the view that AI-based educational support is more effective when designed as a process-oriented scaffold rather than as a direct answer-delivery tool (González-Ortiz-de-Zárate et al., 2025). In the present study, the intervention did not simply increase access to information. Instead, it externalized key regulatory steps into structured, repeatable actions, which likely reduced the cognitive and emotional burden of learning for students facing chronic contextual constraints. This interpretation is also consistent with prior work suggesting that underachieving students often struggle less with content availability per se than with learning initiation, continuity, and post-failure recovery under pressure (Lewis et al., 2016).

From a mechanistic perspective, the results are consistent with an SRL process-chain account. The intervention first supported students in converting vague intentions into specific and feasible plans, then reinforced execution through strategy prompts and monitoring cues, and finally strengthened reflection and attributional adjustment through guided review (Feng et al., 2025). The mediation analyses further support this interpretation by showing that SRL functioned as a proximal pathway linking intervention exposure to academic and behavioral outcomes. In practical terms, this means that improvements in test scores and classroom engagement were not isolated effects, but were embedded within broader changes in how students organized, enacted, and sustained learning. The temporal pattern of findings is also important. Process-level improvements in SRL appeared to precede or accompany later gains in academic indicators, which reinforces the value of evaluating educational interventions through both outcome measures and process-sensitive indicators rather than relying on grades alone (Fong et al., 2023).

The follow-up findings provide additional nuance. Although intervention effects remained significant at T2, the adjusted between-group difference in SRL total was slightly smaller at follow-up than at post-intervention, indicating partial attenuation after the intensive support phase ended. This tapering is not unexpected for habit-oriented interventions and should not be interpreted as evidence of intervention failure. Instead, it suggests that SRL gains were partly maintained but remained sensitive to reduced scaffolding intensity. The Tier-3 growth-oriented support pathway is relevant here, as it was designed to facilitate maintenance through adaptive challenge, mastery-oriented feedback, and restart cues after interruption. Together, these results suggest that SRL in this context functioned not only as a mediator of short-term gains but also as a partial sustaining mechanism, while also indicating that longer-duration support or booster phases may further strengthen maintenance (Azevedo et al., 2023).

With respect to educational equity, the intervention showed a compensatory pattern. Students with greater socioeconomic disadvantage, lower baseline SRL, and more severe prior academic risk tended to benefit more from the intervention, whereas effects did not differ meaningfully by gender or grade level. This pattern suggests that the intervention did not simply magnify pre-existing advantages. Instead, it provided frequent, low-burden support in a form that was usable by students with higher baseline vulnerability. The modestly stronger effect observed among students with constrained digital access is also notable and is consistent with the intervention’s low-threshold, context-adapted design. At the same time, these findings should not be taken to imply that AI alone guarantees educational equity. The intervention was effective in part because it explicitly incorporated real-world constraints, micro-strategy support, and safety boundaries, and because it was implemented within a supervised school-based structure rather than as an unsupervised technology deployment (Miao and Holmes, 2023).

An important limitation is that the intervention combined AI mentoring with teacher and research-assistant oversight, and these human support elements may have contributed to observed gains. Although the AI mentor was the primary delivery interface for planning, prompts, and reflective guidance, students also operated within a context of visible adult supervision, risk monitoring, and occasional human check-ins. This means the effects observed here cannot be attributed exclusively to AI-generated guidance in isolation. The supportive presence of teachers and trained supervisors may have reinforced accountability, emotional safety, and sustained engagement, particularly for students with higher stress or irregular study conditions. This point is especially relevant when interpreting the intervention’s equity-related effects. The combination of structured AI scaffolding and bounded human oversight may be the active ingredient in real-world implementation, rather than either component alone. Future work should therefore compare different supervision intensities and explicitly test how human check-in frequency interacts with AI-supported mentoring processes and outcomes (Raposa et al., 2019).

This study also contributes to ongoing debates about the risks of generative AI in education. Prior concerns have emphasized that AI use without guardrails may encourage dependency, shortcut behavior, or superficial task completion. The present findings suggest a more differentiated interpretation. When AI is embedded within a constrained, process-focused design that prohibits answer substitution, emphasizes strategy and reflection, and operates under clear safety and escalation rules, it can support learning regulation rather than displace it. In this sense, the educational value of generative AI may depend less on the model itself than on the pedagogical architecture, governance rules, and implementation context in which it is used. Future research should extend this work through longer follow-up periods, finer-grained modeling of dialog and process-log trajectories, and cross-context testing across subjects, schools, and digital-access conditions, while also examining how AI feedback style and human oversight jointly shape long-term maintenance and equity effects.

## 5 Conclusion

This study demonstrates that, for socioeconomically disadvantaged students with persistent academic underachievement, integrating AI-supported e-mentoring with structured self-regulated learning (SRL) training can strengthen core learning-process competencies and, in turn, support improvements in academic performance, learning engagement, and learning persistence. The observed effects are consistent with an SRL-based mechanism chain, in which process-level competencies serve as a central leverage point linking the intervention to both academic and behavioral outcomes. The findings also indicate a compensatory pattern: students with higher baseline risk and fewer available resources tended to benefit more from the intervention, highlighting its equity-oriented potential. Within a school-based framework that combines teacher oversight with clearly defined safety procedures, positioning AI as a scalable, process-oriented scaffold rather than a direct answer-providing tool may offer a low-burden and sustainable form of support for disadvantaged learners. This approach may facilitate the gradual development of stable learning habits and contribute to reducing persistent educational inequality over time.

## Data Availability

The raw data supporting the conclusions of this article will be made available by the authors, without undue reservation.
